# Carer preferences of route of administration of transmucosal diamorphine and willingness to take part in a randomised controlled trial: an interview study (DIPPER)

**DOI:** 10.1186/s12904-022-00951-2

**Published:** 2022-05-17

**Authors:** Liz Jamieson, Emily Harrop, Christina Liossi, Katherine Boyce, Lorraine Mitchell, Margaret Johnson, Yogini Jani, Victoria Akinyooye, Simon S. Skene, Ian C. K. Wong, Richard F. Howard, Kate Oulton

**Affiliations:** 1 Centre for Medicines Optimisation Research and Education, University College London Hospitals NHS Foundation Trust & UCL School of Pharmacy, London, UK; 2 Helen and Douglas House Hospice, Oxford, UK; 3 School of Psychology, University of Southampton, Highfield, Southampton, SO17 1BJ UK; 4 Great Ormond Street Hospital Children’s Charity, London, UK; 5 Patient and Public Representative C/o Department of Public Health and Primary Care, University of Cambridge, Cambridge, UK; 6 Surrey Clinical Trials Unit, University of Surrey, Guildford, UK; 7 Great Ormond Street Hospital for Children, NHS Foundation Trust, London, UK

**Keywords:** Paediatrics, Palliative care, Terminal care, Opioids, Diamorphine, Breakthrough pain, Pain management

## Abstract

**Background:**

Children and young people are usually given liquid morphine by mouth for breakthrough pain, which can take thirty minutes to work. A faster-acting, quickly absorbed, needle-free pain medicine, that is easy to administer is needed such as transmucosal (sublingual, buccal, intranasal) diamorphine. Research evidence relating to the administration of medication for breakthrough pain in children and young people is limited. This study aims to describe the experiences and preferences of parents and/or children and young people regarding the route of administration of diamorphine, barriers and facilitators comparative to oral morphine, and participation in a randomised controlled trial.

**Methods:**

In-depth, semi-structured interviews with parents and/or children and young people at home or hospital/hospice.

**Results:**

Thirteen interviews with: nine mothers, one father, and three sets of parents jointly. No interviews took place with a child/young person. Most families had experience of the buccal route which was effective in ease of administration and time to control pain. The intranasal route was preferred by parents irrespective of experience. Parents’ willingness for their child to take part in a trial depended on the time commitment, their child’s pain trajectory and the stability of analgesic requirements.

**Conclusion:**

A randomised controlled trial of oral morphine versus transmucosal diamorphine would need to consider trial logistics, especially time commitment. Parents felt that the trial should be introduced initially by the clinical team, with written information from the research team, and sufficient time to ask questions. Patients who had discontinued oral morphine because of side effects, or those with gastrointestinal failure, should be excluded. Maintaining stability in pain management was essential to families, so the timing of the trial is a potential issue.

## Background

The number of children and young people (CYP) with life-limiting and life-threatening conditions in England rose from 32,975 in 2001/02 to 86,625 in 2017/18, partially attributed to by increased survival and earlier recording of such diagnoses [1]. These CYP have unique palliative care needs, including the management of distressing symptoms.

There is currently no fast acting, safe medicine licensed for managing breakthrough pain in babies or, CYP who receive regular analgesia as part of their palliative care. Needle phobia means that CYP and parents often avoid asking for extra medicine, so a needle-free preparation that is easy to administer at home/place of choice, is needed. Oral morphine is the standard therapy for breakthrough pain in CYP in most patient groups (for community palliative care). However, it can take up to thirty minutes to work. Diamorphine can be easily given transmucosally (under the tongue, up the nose, or into the cheek pouch), where the rich blood supply absorbs it rapidly, working almost as quickly as an injection. Diamorphine is relatively potent and very soluble, making it a good choice for transmucosal use. Buccal or nasal administration is felt to offer rapid, needle-free analgesia. Diamorphine could be used sublingually or buccally. The Association for Paediatric Palliative Medicine (APPM) formulary is used for dosing and information is available for both diamorphine transmucosal administration and oral morphine [2].

Literature reviews and clinical trial registry searches did not identify any randomised controlled trials (RCT) in paediatric palliative care for breakthrough pain; hence the feasibility and practicality of conducting an RCT in this area remains unknown. Barriers to such trials may include recruitment issues around acceptability of study designs, gatekeeping due to perceptions of patient/family vulnerability, and logistical issues reducing access to participants [3].

The paucity of research evidence on medication for breakthrough pain in CYP prompted a specific research recommendation ‘what is the acceptability, safety, and effectiveness of different types of opioid analgesia for breakthrough pain in children and young people with life-limiting conditions who are having end-of-life care in the community [4]?’.

**Fig. 1:**
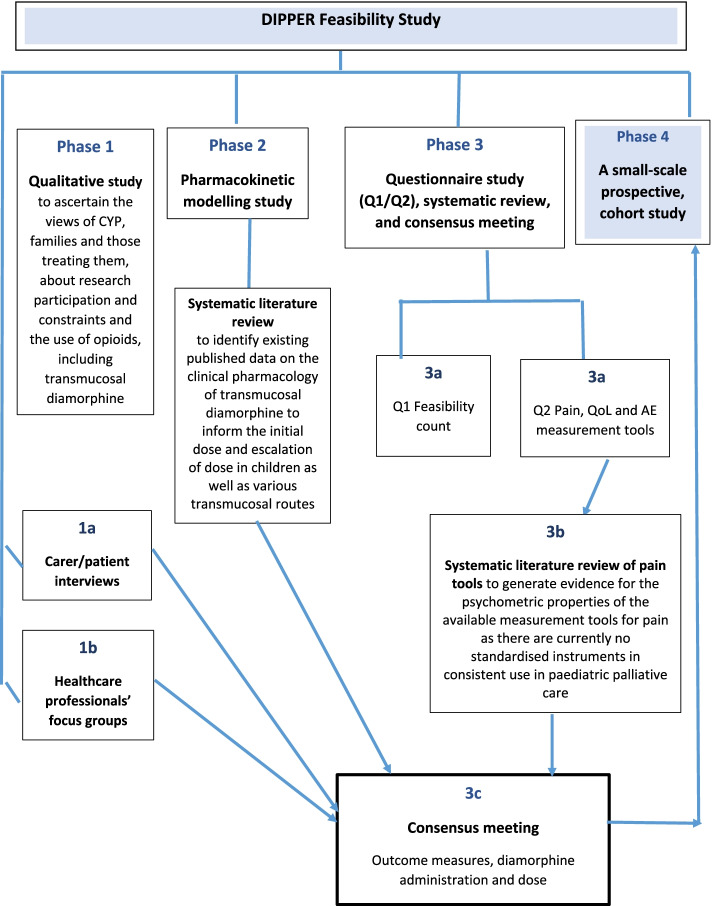
Phases of the DIPPER study (funded by the National Institute for Health Research (NIHR) Research for Patient Benefit (RfPB) Programme (Grant Reference Number PB-PG-0317-20036))

The method of administration of any pain medication must be acceptable to patients and their parents, and be safely delivered in realistic dose increments, without the need for specific professional training. This paper reports the findings of Phase 1a, an interview study as part of a wider 4-phase feasibility study of a randomised controlled trial (Fig. 1).

The aim was to describe the experiences and preferences of parents and/or CYP regarding:

1. the most acceptable route (buccal, sublingual or intranasal) for transmucosal diamorphine;
2. the barriers and facilitators of transmucosal diamorphine and oral morphine use;
3. the barriers and facilitators to participation in a trial including randomisation.

## Method

### Study design

A descriptive, cross-sectional study comprising in-depth, semi-structured interviews with parents and/or CYP.

### Sampling and recruitment

#### Sampling strategy

The purposive sampling strategy was designed to include a sample of CYP aged 2–18 years with a life-limiting condition receiving palliative care and requiring strong opioid analgesia both for background *and* breakthrough or incident pain, and their parents. Parents and CYP who did not understand spoken English were excluded unless a suitable translator was available, as were those lacking capacity to make an informed decision about participation.

Families under the care of palliative care services were recruited from eleven participating centres (hospices and hospitals) in different geographical areas, regardless of location of care (community, hospice or hospital). With a small target population, our aim was to recruit 2 families per centre providing diversity and increasing generalisability of findings.

#### Recruitment

Potential families were approached by the clinical team, who introduced the study and provided study packs, including participant information sheets. Participants were allowed as much time as they wished to consider the information and one reminder was given. Families interested in taking part contacted the researcher who answered any questions and, for those who agreed, arranged the interview.

### Data collection

Semi-structured interviews were conducted by LJ, an experienced qualitative researcher, using an interview guide developed using relevant literature, the teams’ experience and feedback from patient representatives who also piloted it to ensure face validity. For clarity, breakthrough pain was described to families as ‘extra’ pain requiring additional medicine on top of medicines given to control existing baseline pain.

A choice of location (family home, hospital, hospice) was offered for the interview except during Covid-19 when interviews could only take place over the telephone. Written consent from the parent (with assent from the child if appropriate) was obtained by LJ. Interviews were audio-recorded with participant’s permission. In the case of face-to-face interviews brief notes were taken by research assistant VA who was also present. A £20 gift voucher was given in recognition of participants time given, although they were not informed beforehand. Basic clinical information was provided to the researcher by the clinical team after obtaining consent.

### Data processing and analysis

Interviews were transcribed verbatim and analysed thematically [5], using NVivo software (QSR International (UK) Limited, Southport, UK) by LJ and KO. A description of each stage of data analysis is provided in Table 1.

**Table 1:** Data analysis

Stage	Description
Familiarisation	Each transcript read multiple times, anonymised Recordings re-listened to as needed
Identifying thematic framework	Key themes and issues coded by LJ with a sample coded by KO using interview topic guide as a starting point The initial coding framework was used to code subsequent transcripts, and new codes were added as they emerged using the constant comparative technique. This involved the continual appraisal of items in the dataset to identify and explain differences and similarities in the experiences and perspectives of different participants
Indexing	Annotating transcripts for consistencies
Charting and mapping	Rearranging data and framework to generate order
Interpretation	Detailed exploration of revised thematic framework

Throughout the data analysis, the team discussed and reviewed emerging themes until consensus was reached. The final themes were presented to the PPI group to ensure interpretations remained close to the direct experience of families

## Results

Fifteen parents returned the reply slip within the recruitment period (October 2019—July 2020). Two reply slips were received in the post after lockdown ended, but it was too late to interview these families who were contacted to apologise. All those who returned the reply slip or contacted the researcher were interviewed, with the exception of one child who passed away prior to the interview and one parent who was uncontactable. A few families refused as they felt fully committed in their caring role, despite wishing to participate, and in a few cases site staff judged that eligible families were under too much pressure and did not give out the information pack.

LJ conducted interviews with 13 families (9 mothers, 1 father, 3 sets of parents jointly) from 7 of the 11 participating sites. Four took place in the family home; three at the hospice or hospital and the remainder by telephone/video. Interviews lasted between 40 and 75 min. There were no interviews with a CYP; one passed away prior to interview and three who were eligible declined but provided verbal assent for their parent to take part.

Of the 13 CYP who were the focus of discussion, six were male and seven were female. Five young people were older than 14 years, five were aged between 8 and 13 years old and three were 2–7 years old, with an age range from 3 to 17 years. Five were recruited from hospitals and eight from hospices.

The majority of the patients (*n* = 8) had progressive neurological diagnoses, one patient had an oncological diagnosis, two patients had static brain injuries and two had genetic skin conditions. Four patients were verbal.

### Experience of breakthrough pain

Sources of pain were varied including stomach/bowel, bone and blistering skin causing pain on movement. Nine children (69%) were non-verbal/end of life and their inability to communicate was a challenge for parents in deciding when they were in pain and pain location. There was difficulty in distinguishing ‘pain’ and ‘distress’. Specific physical and verbal signals, including sweating, grimacing, contortions and crying, were interpreted by parents as signs of distress. Parents felt that they knew their child well enough to read these signs, but that this was not always the case with paid carers or healthcare professionals. They reported that aggressive behaviour was sometimes not understood by healthcare professionals as an indicator that the child was in pain:

“Right at the moment, (X)’s uncomfortable at the moment, so this is (X) in distress, but he’s not, this noise is he’s got tummy ache or bowel pain”. (003)

“So he can grab sometimes with anxiety and pain but when he’s in the breakthrough, just the breakthrough pain and he’s not managing it he teeth grinds. He doesn’t do that at any other time, doesn’t do it when he’s anxious, he doesn’t do it when he’s sad, he doesn’t do it when he’s excited, he only ever does it when he’s in pain”. (005)

The number of episodes of breakthrough pain experienced was extremely variable, both across CYP with different conditions, and for the same child.

“… if he’s having breakthrough pain he’d probably have about maybe fifteen sprays throughout the whole day … depending on what his pain is and then he can go days where he has none. But not necessarily the full amount of fifteen, it could be less than that…”. (005)

“So how many times a day would she have Oramorph for breakthrough pain? On a bad day? Up to six times. Yeah, on a very, very bad day. So might there be some days when she doesn’t have to have any breakthrough? Yeah, you know, currently we’re, you know, we’re not having any”. (012)

### Management of pain medicines

Nearly all parents were responsible for administering medicines to control their child’s pain, being given repeatedly during the day/night. Several showed the researcher the medicines laid out for the day demonstrating the complexity of the regimens. Nine patients received medicines via gastrostomy/jejunostomy, two via parenteral nutrition and two oral. Most families were extremely knowledgeable about their child’s pain and medicines. A few families had only recently been introduced to opioid medicines, either during end-of-life management or at crisis points, and they had considerably less experience and confidence in the use of pain medicines:

“To be honest they were very good in telling us what they were doing. We were probably not very good in absorbing it all. It was all just … too much for us”. (007)

Additional challenges included paid carers not always being allowed to prepare medicines, and restrictions at schools; with staff being unable to administer certain pain medicines or make decisions on pro re nata **(**PRN) medicines.

“…to be fair they’re [school staff] not medically trained”. (002)

Other issues included parents making up complicated preparations, managing shortages of supplies and keeping drugs charts updated. However, keeping their child free from pain was their biggest challenge:

“… making sure that (X) has a good quality of life and is not left in pain. That’s my biggest challenge”. (001)

Parents expressed a fear of under or overdosing their child explaining how they aimed to stay within maximum daily allowances, but having to balance not waiting too long and the pain escalating:

“I always feel a bit worried about the amount of pain relief he has, the side-effects from that … And when we question those days like ‘oh does he definitely need it?’ we can then miss that window and he can end up being in so much pain that we struggle to get back on top of it”. (005)

“Before the patches, the challenge was to keep to the time limit”. (012)

Parents were well organised in relation to the daily routine of managing medicines for their child’s pain, and minimising the risk of errors:

“I have to be 100% completely organised because then I know there’s not going to be any errors with her medication”. (001)

They also found it helpful to share the medicine management role with their partners, and all appreciated support from pain and palliative care teams, local community nursing teams and pharmacists; particularly those whose first experience of the hospice teams were in crisis situations. Although some parents said they had a good relationship with their GP, GPs admitted to them that they did not have the relevant specialist experience.

### Experience of routes of administration

Liquid oral morphine (Oramorph®) was frequently administered for breakthrough pain, although several patients had discontinued this due to a lack of effect, gastrointestinal failure or side effects. Experience of diamorphine was limited, with four CYP having had buccal diamorphine, and four an unlicensed intranasal diamorphine preparation (Ayendi, now withdrawn). Most parents were familiar with the buccal route with experience of administering buccal midazolam (a benzodiazepine anticonvulsant) but limited experience of sublingual administration. The intranasal route was limited to the flu vaccine or Sinex/saline sprays.

### Facilitators and barriers to using oral morphine and transmucosal diamorphine

**Fig. 2:**
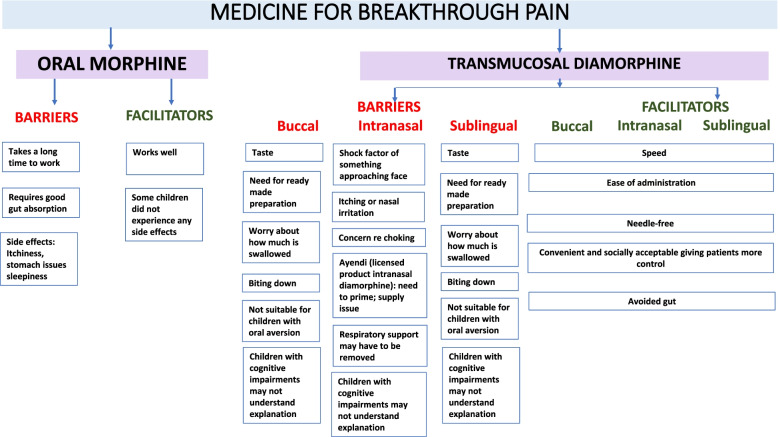
Thematic Analysis: barriers and facilitators of oral morphine and transmucosal diamorphine

Figure 2 shows the main facilitators and barriers to oral morphine and transmucosal diamorphine.

Whilst oral morphine was said to work well, it took anything from 20 to 60 min to act, by which time the child’s pain had escalated. Nevertheless, this was still faster than some other common pain medicines, such as paracetamol or ibuprofen:

“She prefers to take the Oramorph if she’s having breakthrough pain, because she says it works faster than the Paracetamol and the Ibuprofen, yeah, and it’s more effective…”. (002)

Another barrier to oral morphine use was the need for good gastrointestinal absorption:

“We never know, really, how much she’s absorbing. So we assume she absorbs, but it would work better sometimes than it would other times”. (009)

“I really do think that Oramorph is a really good drug. And I think, obviously our issue with [X] is that her absorption is a bit slow. But, you know, in a typical child, if you like, and if their absorption was better, then Oramorph would work a lot quicker than it does with [X]”. (010)

Side effects reported included itchiness, stomach pain and sleepiness, thus impacting on quality of life:

“I don’t just want to hit her with Oramorph every time because then she’d spend all her time asleep and then coming back to the quality-of-life issue, she’s not really got much quality of life if she’s just asleep all day because she’s dosed up on Oramorph”. (001)

“He got a lot of stomach pain with it. So whilst when we gave it him, initially we started to see a little bit of reduction, he’d start clutching and holding his tummy, so it was almost like we were relieving one pain to give him another set of pain…” (005)

In terms of transmucosal diamorphine, ease of administration and speed of action, ranging from instant to ten minutes, were reported by parents as benefits of all three routes of administration:

“Yeah, I think it (buccal) just absorbs and works so quickly”. (010)

“I think the nose is easier to get to than the mouth, there’s less chance, really, in terms of if the child decides they just don’t want that medicine, although you might have to hold them down, you will be able to put it in and you will know you’ve given the dose and, you know, so I think it’s probably more secure in terms of knowing that a certain amount has been given”. (011)

One family said that the speed of intranasal diamorphine allowed her child to make a connection with the spray:

“It starts to work within seconds. And he, otherwise he wouldn’t know that it helps and he wouldn’t let us carry on doing it because he wouldn’t see the point. He’s a very logical little boy” …”he used to crash his trains and then say, ask for the spray to fix his trains”. (005)

Other perceived benefits of transmucosal products included the fact that they were more convenient and socially acceptable, giving carers and/or patients more control and improving their quality of life. They also avoided both the digestive system and the use of needles:

“It’s been life changing for him, like from a quality-of-life point of view. We can go out with him and take him out knowing that we can just get something and give it him quickly for his breakthrough pain, instead of then having to find a sterile area to then draw up something, to then administer it IV”. (005)

“It’s much more helpful to have the nasal spray or the midazolam or when we were giving the fentanyl sublingually it was much quicker and easier to administer those when you’re out and about because you can just have them in your handbag…”. (006)

The buccal and sublingual routes which require access to the mouth were said to have many of the same drawbacks as oral administration, which included patients biting down and parents worrying about how much was swallowed.

“The other thing about the buccal is always the worry of has it, because the nature of how it’s absorbed, you know, they don’t need to swallow it, it just goes into the cheek, but you’re always wondering whether has some spilt out and, you know, has that affected how much he’s had or has he swallowed some”. (011)

One parent, whose child produces a lot of secretions when they have a seizure, suggested that it would be helpful if the liquid was coloured because it would enable her to tell if it the buccal midazolam had been fully absorbed or has “dribbled out” *(013).*

Taste was another issue that was felt to be off-putting for patients:

“For her buccal wouldn’t work. [She] does not like the taste of anything really sweet, so all she’ll drink is water, she won’t drink juice at all”. (009)

“I don’t know what Oramorph tastes like, but I’ve never given it to him orally, it’s always been through his gastrostomy. But if it doesn’t taste good, it would make him vomit”. (013)

Further disadvantages of the buccal and sublingual routes were lack of suitability for CYP with oral aversion and those on oxygen using continuous positive airway pressure. Also, CYP with cognitive impairments may lack the necessary understanding:

“We can’t explain about what we’re doing to him, that we’re going near his mouth or that we’re putting something into his mouth. Also that he wouldn’t bite or that he wouldn’t thrash around. So it makes it really difficult for us to do anything buccal with (X)”. (005)

One drawback of Ayendi, a licensed product of intranasal diamorphine for use in emergency settings, was the need to prime the product (by holding the bottle upright and actuating eight times to achieve the optimum spray before the first use). Itching was reported as a side effect, as was possible nasal irritation. Several families mentioned a supply issue:

“So if it would be a spray then if the children keep their head moving they could find it difficult to get it through”. (008)

Some parents were also concerned that their child might be frightened by something approaching the face (“shock factor”). Again, there was a concern that some patients might choke.

A major disadvantage of transmucosal products was the need for ready-made preparations:

“I need it all to be there and ready so we can be responsive to pain immediately”. (001)

### Preferred routes of administration

The intranasal route was preferred by the majority of parents (*n* = 8) with and without experience of the route.

“If I were to go for one I’d go for the nasal one, only because I think you’ve got less risk of him choking or him swallowing it straightaway”. (003)

“..so certainly for us the nasal option seems to be the most effective”. (006)

Three parents preferred the buccal route.

“.. a lot of people are familiar with buccal midazolam, a lot of children have seizures and for a lot of children that rescue medication is buccal midazolam, so you’re starting from a point where you actually know where the buccal area is, how you put a syringe into the buccal area because most parents will have done that with Buccal midazolam”. (001)

One parent said she would prefer a rectal suppository for her child than any of the proposed routes,

as she did not feel it was as invasive as the mouth/ nose area.

Five parents said their least favourite route was sublingual:

“Probably under the tongue because I think that would be the hardest to try and achieve, I think it’d probably easier to get a syringe in-between your gums and the side of your mouth is probably easier than trying to get it under his tongue, I think under his tongue he would hate”. (003)

“I haven’t had any experience of that but what I think is that if it’s like the lingual one that they could like resist it with their tongue. Yeah, because at time she won’t open her mouth or she would like gnarl with her teeth..”. (008)

One parent said their least preferred route was intranasal:

“I just think that she wouldn’t be happy, I think it would be a bit of a shock to her, having something up her nose”. (010)

However, it was also recognised that “it’s obviously going to be different for different children”. *(011).*

### Barriers and facilitators to taking part in a trial of transmucosal diamorphine and randomisation

**Fig. 3:**
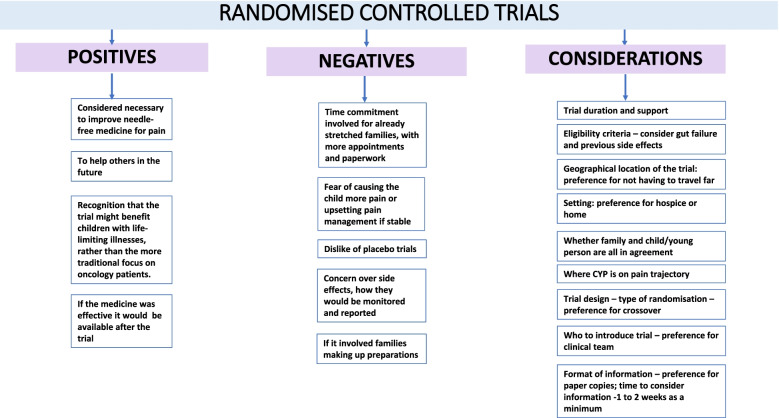
Thematic Analysis: barriers and facilitators to taking part in a randomised controlled trial of oral morphine and transmucosal diamorphine

Figure 3 shows the main barriers and facilitators to taking part in a trial. Most families had heard of clinical trials, but few had taken part in one. All appreciated the benefit of a proposed trial; to improve needle-free pain medicines, as families agreed that: “the last thing any parent should see is their child in pain”. (012).

There was recognition that a trial might benefit CYP with life-limiting illnesses, rather than the more traditional focus on oncology patients.

“As parents of children with complex needs…unfortunately we’re not the first ones in this situation and we’re not going to be the last ones….if people don’t start doing these things, we can’t help people that are coming along as well”. (013)

Disadvantages of taking part in trials included another time commitment for families with “an already very complicated life” (001), with more appointments and paperwork. However, the time commitment did not put some families off, particularly if they felt it “helped coming children in the future” (012).

*A* fear of causing the child more pain was also reported:

“Well, I think the only disadvantage would be if you were quite settled and then for some reason that new method didn’t actually work as well, and it, you know, it allowed the child to be in more pain”. (011)

Concerns about the clinical stability of their CYP was the main reason parents gave for not allowing their child to take part in a trial, particularly if it had taken some time to reach that point:

“I think he’s just been through so much hasn’t he in the last nine months? And we’ve just got to a point where something’s working”. (005)

“... I think, for us it would literally be where [X] is at that point that you wanted her to take part in the trial, does that make sense?” [010]

The geographical location and setting of the trial were said to be important, with families expressing a preference to take part in a hospice, which was felt to be more relaxed, or at home, to avoid having to travel.

..”for us, you know, geographical location is an issue. So, you know, if somebody said to us, can you come to London every week for the next 16 weeks, we’d just say no, because that’s too big an impact on our lifestyle. But... I suppose if we felt that it was going to make a massive difference then maybe we would consider overruling that but I doubt it”. (006)

Some families referred to placebo trials, which they would not want to take part in:

“My reluctance would be if it was a double-blind trial and it was Diamorphine or placebo, I wouldn’t want to take part in that because I don’t want to see my child suffer and I think that must be a very difficult one to opt to take part in, I’m sure people would, personally I probably wouldn’t. But if it was a case between this pain relief or that pain relief...” (001)

Having to make up preparations would also be off-putting for families:

“that would be the thing that would be prohibitive, if it was not prepared for me and it was timely to be drawing up ... if it came as a liquid and I could just draw it up out of a bottle immediately, like I do with buccal midazolam, that takes seconds, that’s not complicated. It’s the whole needle and syringe and ampules and all of that nonsense that just takes time to make sure you’ve got it right”. (001)

It was clear that the child and family must all agree about participation:

“If (X) didn’t want to. That would, yeah, because she’s (a teenager), and we let her, we have to let her take decisions about her own healthcare, because otherwise she won’t go along with whatever we’re suggesting, you know, so it’s got to be her decision”. (002).

One family expressed a general fear about a different route of administration:

“I would just be nervous about it, but I think it’s more the delivery method for me, if you said you could put it down his PEG, or you could give it as a suppository, because it’s a medicine that we’re familiar with that route, I think it’s the route that makes me nervous”.(003)

A few families said that nothing would stop them taking part.

“I mean, as long as I knew that he was actually getting treatment, then absolutely nothing, you know…” (013)

“.. I think most people would say “if there’s some way that I can help then yes I will”. (007)

### Information for families

When making their decision about whether to give permission (consent) for their CYP to take part in a trial, parents reported they wanted to have a clear aim of the study, information about possible side effects and how these would be monitored and reported. Families wanted to know how long the trial would last and what support would be available. They would seek reassurance that their child would not be left in pain and that the medicine, if found to be effective, would be available after the trial.

“… I suppose if we were put onto the part of the trial that was trying Diamorphine and we found it to be significantly less effective for her pain than Oramorph then we would want to end our participation, rather than see her in pain, but I think you’d agree that it would be unethical to continue”. (002)

“.. I’d want to know what the side-effects are…”. (003)

“I think you’d want to know what it was all for, you know, so what you were hoping to achieve with the trial …..if it seemed to work, for example, would you then be on the first list to receive it … and I think the other business about, you know, information security sort of thing, because I think that that’s where there was a lot of explanation about that, how your information will be used and stored …”. (011)

### Randomisation

The understanding of ‘randomisation’ was varied. A crossover trial, where patients trial both oral morphine and transmucosal diamorphine in a randomised order, was felt by most to be preferable to simple randomisation, whereby a patient would be allocated to receive either the current recommended treatment for breakthrough pain (oral morphine) or another (transmucosal diamorphine).

“…. I’m presuming what you would do is, one would be as we would do normally, but it may or may not contain the Morphine, and then one would be either the spray or buccal that we’d be told to give. And then, but he would still receive the pain relief, we just don’t know which delivery, so that’s fine”. (013)

“Trialling them both, because then I could prove that we knew what we were talking about [laughs]. Even if you didn’t know which one was active? I’m confident that I could tell which one. Yeah, because of the way they work on [X], you know”. (014)

### Introducing the trial to families

Parents felt that the trial should be introduced initially by the clinical team, with written information from the research team, and sufficient time to ask questions. They felt that the information for the interview study was very clear, not too long, and they appreciated being given full information about what would happen and, specifically, how the data would be used.

Parents differed in their thoughts about when the trial should be introduced, with some saying at the beginning, after diagnosis, when there are often pain control issues and others saying it was better when the child was stable and the parents were less stressed. Other parents felt they would not want their child to take part if they were comfortable for fear of upsetting their stability. Some felt that any time was good, as the parents could always say whether or not it was a good time for them, in other words, allow people to make their own decisions.

“ well, that’s a difficult question [when is a good time to approach families], I think in some ways you’ve got to just approach them, but allow people to say, “Actually, right now it’s probably not right for me to be part of it”. (011)

“We’re here [in the hospice] 24 hours a day, it’s kind of something to do in a way. We’ve got nothing else to do. Something to occupy your mind for right now sort of thing”. (007)

Families also differed regarding what they felt would be sufficient time to consider the information before deciding to take part in a future trial, with a few needing a longer time to discuss it with their family (4–6 weeks) and the majority saying 1 to 2 weeks was sufficient. Two families felt that it would be important not to be too long.

“.. I think you have to be really careful, because if you leave it too long there’s so many things that happen in life that then take over .., not because they don’t think it’s important or valid, but just sometimes if you don’t just do something when you need to do it, it doesn’t happen. And a week is, I think, just long enough for people who are really busy but are keen, and not too long so that people just don’t bother, yeah”. (013)

All agreed, without hesitation, that they would give permission for the use of their child’s information (data) in the DIPPER trial.

## Discussion

### Main findings

The interviews aimed to determine the most acceptable route (buccal, sublingual or intranasal) of administration of transmucosal diamorphine. There is often concern about addiction to opioids, but addiction is very rarely encountered when opioids are used responsibly in the context of palliative care, with appropriate opioid stewardship [6]. Nasal, buccal and sublingual routes of transmucosal administration of diamorphine are all possible, and use of nasal diamorphine for traumatic long bone fractures in healthy children is widespread practice in the UK.

Buccal was the most familiar route, primarily from using buccal midazolam for seizures; consistent with previous findings from focus groups involving healthcare professionals [7]. Even though fewer people had experience of the intranasal route, it had greater appeal, largely because it avoided problems of administering via the mouth, such as the child biting down and vomiting, and it was felt to be easier and quicker to administer via the intranasal route. Parent representatives also favoured the intranasal route at a consensus meeting. Speed of administration and time to pain relief were really important factors. Anything that involved complicated preparations was not welcome as they wanted something to use “in the heat of the moment”. Factors to consider regarding the introduction of transmucosal products included: school policies, as several of the children were attending school; carers coming into homes who may not always be able to make up medicines and having ready-to-use preparations, the latter being particularly relevant in the light of the current pandemic, with nurses unavailable to go into family homes to administer medicines. A recent editorial about families administering end-of-life drugs at home during the crisis, acknowledged that the buccal and sublingual routes are less commonly used with evidence coming primarily from professional experience and paediatric palliative care [8].

A survey study assessing acceptability of nine routes of administration of analgesia for the treatment of mild/moderate and severe breakthrough pain involving one hundred adults with cancer-related pain referred to a specialist palliative care unit, found that the acceptability of the different routes varied, and appeared to be influenced by previous experience or expectation (e.g. taste) of that route and by the severity of the pain [9]. Our study findings are similar. Patients in the survey had less experience with the newer routes of analgesia (i.e., nasal, transmucosal and inhaled) and these were generally less acceptable than the more conventional routes. Reasons for finding these routes unacceptable were not always immediately apparent, e.g., unacceptability related to `fear of a bad taste/nausea’.

Our interviews also aimed to ascertain the acceptability to families of a clinical trial of oral morphine versus transmucosal diamorphine. Although families welcomed the idea of a clinical trial to advance needle-free pain medicines, the timing of the introduction of the trial to families was cited as an overriding issue. Parents were not keen to risk their child’s stability in terms of pain management and would not agree to taking part if their child was comfortable and had taken a lot of time to get to that point. Conversely, if the family was dealing with a crisis, this was also not felt to be a good time. Several families in this study had “moved on” from oral morphine, so perhaps the best time would be when pain medicines are first introduced for breakthrough pain. Parents were generally accepting of a trial, but not one which involved a placebo or if there was a risk that their child’s pain was not controlled, or pain control was lost. Parents preferred the idea of a crossover trial whereby both medicines were trialled rather than simple randomisation. Patient and caregiver concerns over randomisation were also themes found in an interview study of adult patients engaging in palliative and healthcare trials; likewise time constraints for already over-stretched families [10]. Our study also highlighted concerns over the logistics of the trial, such as location and support available.

Most parents felt that the trial should be introduced by the clinical team, which is similar to two studies involving adults [10, 11]. A difference was noted though, with parents’ preference for written information in our study, compared to end-of-life adult patients in the study by Terry et al. who preferred oral information as opposed to a participant information sheet. This may be an important consideration for patients in future trials [11].

Parents were knowledgeable about their child’s medicines and recognised the value and need for research into pain medicines. Phipps et al. found that adult patients who are in more pain may be more likely to participate in research [12]. They hypothesized this may be because patients hope that participation will mean pain issues will be better addressed and this could be a similar consideration for parents of children in pain. Several parents expressed gratitude that this study was taking place and they stressed they would “do anything” when their child was in pain. Parents also felt that they know their child best and understood often unique signs indicating that they were experiencing pain and when they started responding to pain medicines.

A quote from one parent sums up the necessity for researchers to move this work forward to help both the parents and their children with access to fast, effective, safe pain relief:

“…I’m sure all parents would say this, but I would literally do anything for him, but it’s not enough”. (013)

### Strengths and limitations

This is the first study to report families’ experience of the benefits of oral morphine and transmucosal diamorphine for breakthrough pain in children receiving palliative care and highlight their concerns regarding a randomised controlled trial of oral morphine versus transmucosal diamorphine. Recruitment to this study was challenging; partly due to pressures on staff as a result of Covid-19, our eligibility criteria and methodology, and similar studies recruiting at the same time. However, those who were interviewed were very willing to talk at length and in detail. Our feasibility target was to recruit two families per centre. However, even after extending the recruitment time, only 13 families were interviewed, possibly due to different patient populations at sites, our eligibility criteria, and COVID-19. We cannot guarantee that data saturation was achieved but as no new major themes were emerging, we believe we obtained thematic saturation.

## Conclusions

Families welcomed research into pain medicines and expressed a preference for the intranasal route of administration but also agreed that there was familiarity with the buccal route. A randomised trial of oral morphine versus transmucosal diamorphine is possible providing consideration is given to such factors as timing in terms of the patient’s pain management journey, location and setting of the trial, time commitment for families and how the trial is introduced. Although diamorphine is not used in some countries, this could change if the UK were to obtain a licensed formulation.

## Data Availability

The datasets analysed during this study are not publicly available because of the risk that individual privacy could be compromised. All data relevant to the study is contained within the manuscript. Within the limits of confidentiality, more detailed, but anonymous, data may be available from the corresponding author on reasonable request.

## Abbreviations

CYP: Children and Young People
RCT: Randomised Controlled Trial
